# A second monoclinic polymorph of ethyl­enediammonium bis­(hydrogen squarate) monohydrate

**DOI:** 10.1107/S1600536811009019

**Published:** 2011-04-13

**Authors:** Louiza Zenkhri, Thierry Bataille, Nathalie Audebrand

**Affiliations:** a Faculté des Sciences et Technologie et Sciences de la Matière, Université Kasdi Merbah Ouargla, Route Gardaia, Ourgla, Algeria; b Laboratoire Sciences Chimiques de Rennes (CNRS, UMR 6226), Université de Rennes 1, Avenue du Général Leclerc, 35042 Rennes Cedex, France

## Abstract

The title compound, C_2_H_10_N_2_^2+^·2HC_4_O_4_^−^·H_2_O, a new polymorph of ethyl­enediammonium bis­(hydrogen squarate) monohydrate, was synthesized by slow evaporation of an acid solution. The asymetric unit contains two hydrogen squarate anions, two half-mol­ecules of protonated ethyl­enediamine arranged around a twofold axis and one water mol­ecule. In the crystal, N—H⋯O and O—H⋯O hydrogen bonds between the hydrogen squarate anions, protonated N atoms from the amine group and water mol­ecules lead to a three-dimensional framework. In particular, the cohesion between the squarate groups is ensured by very short intermolecular hydrogen bonds bonds. The title compound crystallized together with the previously reported polymorph [Mathew *et al.* (2002 ▶). *J. Mol. Struct.* **641**, 263–279].

## Related literature

For the previously reported polymorph, see: Mathew *et al.* (2002 ▶).
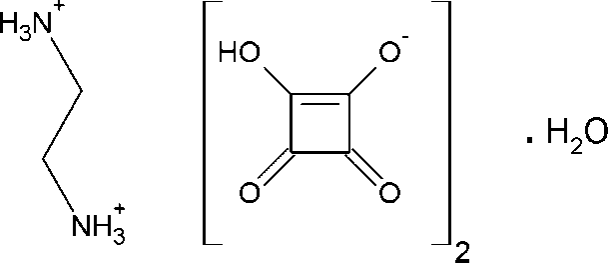


## Experimental

### 

#### Crystal data

C_2_H_10_N_2_^2+^·2C_4_HO_4_^−^·H_2_O
*M* *_r_* = 306.23
Monoclinic, 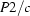

*a* = 14.1907 (3) Å
*b* = 9.0224 (2) Å
*c* = 10.9412 (2) Å
β = 111.789 (1)°
*V* = 1300.77 (5) Å^3^
*Z* = 4
Mo *K*α radiation
μ = 0.14 mm^−1^
*T* = 293 K
0.45 × 0.44 × 0.37 mm

#### Data collection

Nonius KappaCCD diffractometer
16099 measured reflections
2957 independent reflections
2101 reflections with *I* > 2σ(*I*)
*R*_int_ = 0.039

#### Refinement

*R*[*F* ^2^ > 2σ(*F* ^2^)] = 0.044
*wR*(*F* ^2^) = 0.116
*S* = 1.06
2957 reflections
190 parameters
H-atom parameters constrained
Δρ_max_ = 0.28 e Å^−3^
Δρ_min_ = −0.24 e Å^−3^

### 

Data collection: *COLLECT* (Nonius, 2000 ▶); cell refinement: *SCALEPACK* (Otwinowski & Minor, 1997 ▶); data reduction: *DENZO* (Otwinowski & Minor, 1997 ▶) and *SCALEPACK*; program(s) used to solve structure: *SIR97* (Altomare *et al.*, 1999 ▶); program(s) used to refine structure: *SHELXL97* (Sheldrick, 2008 ▶); molecular graphics: *DIAMOND* (Brandenburg & Berndt, 2001 ▶); software used to prepare material for publication: *WinGX* (Farrugia, 1999 ▶).

## Supplementary Material

Crystal structure: contains datablocks I, global. DOI: 10.1107/S1600536811009019/dn2662sup1.cif

Structure factors: contains datablocks I. DOI: 10.1107/S1600536811009019/dn2662Isup2.hkl

Additional supplementary materials:  crystallographic information; 3D view; checkCIF report

## Figures and Tables

**Table 1:** Hydrogen-bond geometry (Å, °)

*D*—H⋯*A*	*D*—H	H⋯*A*	*D*⋯*A*	*D*—H⋯*A*
N1—H1*A*⋯O5^i^	0.89	2.14	2.9205 (17)	146
N1—H1*B*⋯O1*W*^ii^	0.88	1.99	2.8482 (18)	163
N1—H1*C*⋯O8	0.88	1.90	2.7717 (18)	169
N2—H2*A*⋯O2	0.88	1.97	2.8222 (17)	162
N2—H2*B*⋯O1*W*^iii^	0.90	1.94	2.8279 (18)	171
N2—H2*C*⋯O1^i^	0.90	1.92	2.8071 (17)	168
O4—H4⋯O3^iv^	1.05	1.42	2.4675 (15)	179
O7—H7⋯O6^iii^	1.06	1.41	2.4645 (14)	178
O1*W*—H1*W*⋯O6	0.92	2.10	2.8724 (17)	140
O1*W*—H1*W*⋯O8^iv^	0.92	2.40	3.0489 (18)	128
O1*W*—H2*W*⋯O3	0.93	1.88	2.8035 (19)	171

Symmetry codes: (i) 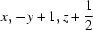; (ii) 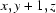; (iii) 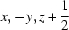; (iv) 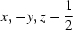.

**Fig. 1.**
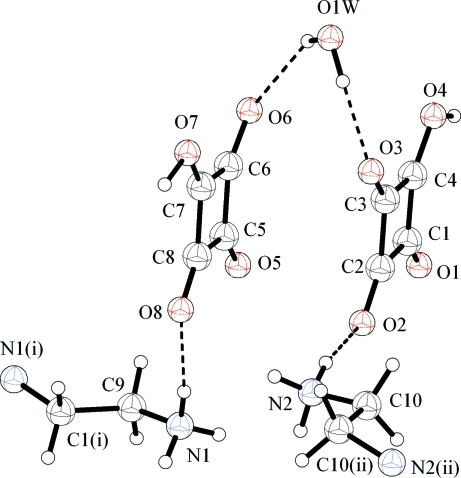
View of the molecular structure of the title compound with the atom labeling scheme. Ellipsoids are drawn at the 50% probability level. H atoms are represented as small spheres of arbitrary radii. Hydrogen bonds are shown as dashed lines. [Symmetry codes: (i) -x+3/2, y, -z+1; (ii) -x+3/2, y, -z]

**Fig. 2.**
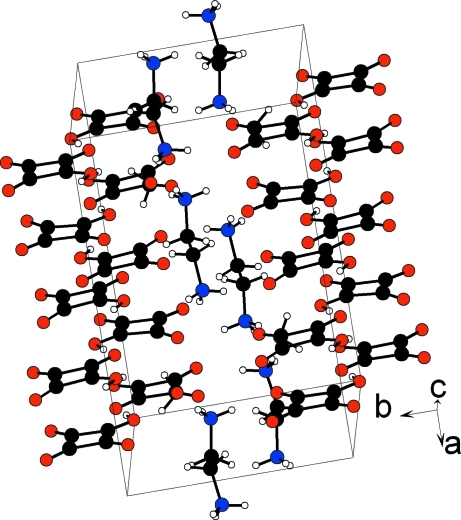
Packing view of the title compound displaying the hydrogen bonds between protonated nitrogen of ethylenediamine, hydrogen squarate and water molecules. H atoms not involved in hydrogen bondings have been omitted for clarity.

## supplementary crystallographic information

### Comment

In the course of a study on mixed squarate of amines and metals, the role of the amine group has been investigated in the topology of the organic-inorganic framework. The preparation did not lead to a mixed compound but to a new hydrogen squarate of ethylenediammonium.

The compound is a polymorph of the compound previously reported by Mathew *et al.*, 2002, whose molecular framework is also stabilized by hydrogen bonds (Fig. 1, Table 1). In the title compound hydrogen bonds connect the hydrogen squarate units along the *a* axis in the form of zigzag chains, which are connected to each other along the *c* axis through hydrogen bonds implying the water molecules, then forming a layer. Amine groups are situated in between neighbour layers and connected to them along the *b* axis through hydrogen bonds leading to a molecular three-dimensional framework (Table 1, Fig. 2).

The main differences between the structures of the two polymorphs reside in the orientation of the amine groups related to that of the mean planes of the squarate groups. Indeed, in the title structure, the ethylenediammonium cations are perpendicular to the squarate groups, while the mean planes between these two molecules in the already reported polymorph deviate to 56.2 (2)°.

### Experimental

The title compound, (HC_4_O_4_)_2_(C_2_H_10_N_2_)(H_2_O) was prepared from an aquous solution (20 ml) of dissolved yttrium nitrate (0.5 mmol), ethylenediamine (0.1 mmol) and 3,4-dihydroxy-3-cyclobutene-1,2-dione, also named squaric acid (0.1 mmol). The slow evaporation at room temperature leads after some hours to the formation of both polymorphs. A metal salt seems to be necessary to the synthesis of the title compound even if its role has not been clearly established.

### Refinement

All H atoms were found from Fourier difference maps but those attached to C and N atoms were fixed geometrically and treated as riding with C—H = 0.98 Å and N—H = 0.87 Å with U_iso_(H) = 1.2U_eq_(C) or U_iso_(H) = 1.5U_eq_(N). The H attached to the water molecule and those of the hydroxyl groups were refined using restraints: O-H= 0.92 (1)Å and H···H= 1.42 (2)Å) for the water and O—H = 1.05 (2)Å for the hydroxyl H with U_iso_(H) = 1.5U_eq_(O). In the last cycles of refinement, they were treated as riding on their parent O atoms.

### Crystal data

**Table:**

C_2_H_10_N_2_^2+^·2C_4_HO_4_^−^·H_2_O	*F*(000) = 640
*M**_r_* = 306.23	*D*_x_ = 1.564 Mg m^−^^3^
Monoclinic, *P*2/*c*	Mo *K*α radiation, λ = 0.71073 Å
Hall symbol: -P 2yc	Cell parameters from 15363 reflections
*a* = 14.1907 (3) Å	θ = 2.6–27.5°
*b* = 9.0224 (2) Å	µ = 0.14 mm^−^^1^
*c* = 10.9412 (2) Å	*T* = 293 K
β = 111.789 (1)°	Block, colourless
*V* = 1300.77 (5) Å^3^	0.45 × 0.44 × 0.37 mm
*Z* = 4	

### Data collection

**Table:**

Nonius KappaCCD diffractometer	2101 reflections with *I* > 2σ(*I*)
Radiation source: fine-focus sealed tube	*R*_int_ = 0.039
horizonally mounted graphite crystal	θ_max_ = 27.5°, θ_min_ = 3.7°
CCD scans	*h* = −13→14
16099 measured reflections	*k* = −11→11
2957 independent reflections	*l* = −18→18

### Refinement

**Table:**

Refinement on *F*^2^	Primary atom site location: structure-invariant direct methods
Least-squares matrix: full	Secondary atom site location: difference Fourier map
*R*[*F*^2^ > 2σ(*F*^2^)] = 0.044	Hydrogen site location: inferred from neighbouring sites
*wR*(*F*^2^) = 0.116	H-atom parameters constrained
*S* = 1.06	*w* = 1/[σ^2^(*F*_o_^2^) + (0.0506*P*)^2^ + 0.4175*P*] where *P* = (*F*_o_^2^ + 2*F*_c_^2^)/3
2957 reflections	(Δ/σ)_max_ < 0.001
190 parameters	Δρ_max_ = 0.28 e Å^−^^3^
0 restraints	Δρ_min_ = −0.24 e Å^−^^3^

### Special details

**Table:**

Geometry. All esds (except the esd in the dihedral angle between two l.s. planes) are estimated using the full covariance matrix. The cell esds are taken into account individually in the estimation of esds in distances, angles and torsion angles; correlations between esds in cell parameters are only used when they are defined by crystal symmetry. An approximate (isotropic) treatment of cell esds is used for estimating esds involving l.s. planes.
Refinement. Refinement of *F*^2^ against ALL reflections. The weighted *R*-factor *wR* and goodness of fit *S* are based on *F*^2^, conventional *R*-factors *R* are based on *F*, with *F* set to zero for negative *F*^2^. The threshold expression of *F*^2^ > 2σ(*F*^2^) is used only for calculating *R*-factors(gt) *etc*. and is not relevant to the choice of reflections for refinement. *R*-factors based on *F*^2^ are statistically about twice as large as those based on *F*, and *R*- factors based on ALL data will be even larger.

### Fractional atomic coordinates and isotropic or equivalent isotropic displacement parameters (Å^2^)

**Table:**

	*x*	*y*	*z*	*U*_iso_*/*U*_eq_	
C1	0.13638 (12)	0.21953 (17)	0.23974 (14)	0.0274 (3)	
C2	0.12945 (12)	0.22646 (17)	0.37292 (15)	0.0294 (3)	
C3	0.13154 (12)	0.06345 (17)	0.37612 (14)	0.0280 (3)	
C4	0.13514 (12)	0.05880 (16)	0.24889 (14)	0.0270 (3)	
C5	0.37318 (12)	0.22227 (17)	0.53401 (14)	0.0282 (3)	
C6	0.37820 (12)	0.05841 (17)	0.53440 (14)	0.0266 (3)	
C7	0.37483 (12)	0.05424 (16)	0.66247 (14)	0.0264 (3)	
C8	0.36698 (12)	0.21411 (17)	0.66793 (14)	0.0285 (3)	
C9	0.45935 (12)	0.62029 (18)	0.68196 (15)	0.0310 (4)	
H9A	0.4620	0.7123	0.6375	0.037*	
H9B	0.4708	0.5391	0.6310	0.037*	
C10	0.00261 (13)	0.36235 (19)	0.68282 (16)	0.0359 (4)	
H10A	−0.0221	0.2683	0.6400	0.043*	
H10B	−0.0408	0.4401	0.6300	0.043*	
N1	0.35844 (10)	0.60417 (15)	0.69124 (14)	0.0330 (3)	
H1A	0.3537	0.6630	0.7544	0.040*	
H1B	0.3127	0.6300	0.6143	0.040*	
H1C	0.3495	0.5112	0.7090	0.040*	
N2	0.10844 (11)	0.38656 (14)	0.69090 (13)	0.0343 (3)	
H2A	0.1155	0.3480	0.6209	0.041*	
H2B	0.1534	0.3450	0.7642	0.041*	
H2C	0.1219	0.4842	0.6931	0.041*	
O1	0.14176 (10)	0.31161 (12)	0.15936 (12)	0.0406 (3)	
O2	0.12416 (11)	0.32661 (14)	0.44601 (12)	0.0469 (4)	
O3	0.13116 (11)	−0.03557 (13)	0.45842 (11)	0.0422 (3)	
O4	0.13646 (10)	−0.05236 (12)	0.17443 (11)	0.0402 (3)	
H4	0.1339	−0.0164	0.0819	0.060*	
O5	0.37214 (10)	0.32156 (13)	0.45711 (11)	0.0411 (3)	
O6	0.38111 (10)	−0.03993 (12)	0.45340 (10)	0.0365 (3)	
O7	0.37617 (10)	−0.05505 (12)	0.73992 (11)	0.0366 (3)	
H7	0.3768	−0.0150	0.8313	0.055*	
O8	0.35656 (11)	0.30475 (13)	0.74692 (11)	0.0430 (3)	
O1W	0.24474 (9)	−0.28124 (14)	0.43628 (12)	0.0436 (3)	
H1W	0.2960	−0.2370	0.4170	0.065*	
H2W	0.2063	−0.2050	0.4510	0.065*	

### Atomic displacement parameters (Å^2^)

**Table:**

	*U*^11^	*U*^22^	*U*^33^	*U*^12^	*U*^13^	*U*^23^
C1	0.0324 (9)	0.0254 (7)	0.0260 (7)	0.0044 (6)	0.0127 (6)	0.0034 (6)
C2	0.0340 (9)	0.0287 (8)	0.0284 (7)	0.0042 (7)	0.0152 (7)	0.0008 (7)
C3	0.0331 (9)	0.0274 (8)	0.0245 (7)	−0.0015 (6)	0.0119 (6)	0.0003 (6)
C4	0.0328 (9)	0.0261 (8)	0.0227 (8)	−0.0002 (6)	0.0110 (6)	−0.0001 (6)
C5	0.0339 (9)	0.0283 (8)	0.0242 (7)	−0.0034 (6)	0.0130 (6)	−0.0006 (6)
C6	0.0310 (8)	0.0284 (8)	0.0228 (7)	−0.0033 (6)	0.0128 (6)	−0.0015 (6)
C7	0.0338 (8)	0.0255 (7)	0.0220 (7)	−0.0013 (6)	0.0128 (6)	−0.0011 (6)
C8	0.0377 (9)	0.0257 (7)	0.0250 (7)	−0.0033 (7)	0.0149 (7)	−0.0019 (6)
C9	0.0310 (9)	0.0318 (8)	0.0315 (8)	−0.0005 (7)	0.0132 (7)	0.0010 (7)
C10	0.0392 (9)	0.0397 (9)	0.0304 (8)	0.0039 (8)	0.0147 (7)	−0.0019 (7)
N1	0.0330 (8)	0.0299 (7)	0.0359 (7)	0.0010 (6)	0.0123 (6)	−0.0002 (6)
N2	0.0462 (9)	0.0273 (7)	0.0354 (7)	−0.0004 (6)	0.0221 (7)	−0.0003 (6)
O1	0.0643 (9)	0.0280 (6)	0.0374 (6)	0.0064 (6)	0.0281 (6)	0.0086 (5)
O2	0.0751 (10)	0.0337 (7)	0.0416 (7)	0.0100 (6)	0.0328 (7)	−0.0038 (6)
O3	0.0740 (9)	0.0308 (6)	0.0279 (6)	−0.0068 (6)	0.0260 (6)	0.0024 (5)
O4	0.0723 (9)	0.0261 (6)	0.0277 (6)	−0.0030 (6)	0.0249 (6)	−0.0033 (5)
O5	0.0632 (9)	0.0314 (6)	0.0341 (6)	−0.0034 (6)	0.0241 (6)	0.0061 (5)
O6	0.0591 (8)	0.0299 (6)	0.0272 (6)	−0.0046 (5)	0.0239 (6)	−0.0059 (5)
O7	0.0646 (8)	0.0243 (6)	0.0283 (6)	0.0017 (5)	0.0260 (6)	0.0031 (5)
O8	0.0776 (10)	0.0254 (6)	0.0366 (6)	−0.0007 (6)	0.0334 (7)	−0.0046 (5)
O1W	0.0384 (7)	0.0416 (7)	0.0464 (7)	−0.0044 (6)	0.0106 (6)	0.0083 (6)

### Geometric parameters (Å, °)

**Table:**

C1—O1	1.2326 (18)	C9—C9^i^	1.508 (3)
C1—C4	1.454 (2)	C9—H9A	0.9700
C1—C2	1.498 (2)	C9—H9B	0.9700
C2—O2	1.2273 (19)	C10—N2	1.487 (2)
C2—C3	1.471 (2)	C10—C10^ii^	1.499 (3)
C3—O3	1.2699 (18)	C10—H10A	0.9700
C3—C4	1.412 (2)	C10—H10B	0.9700
C4—O4	1.2966 (18)	N1—H1A	0.8933
C5—O5	1.2253 (18)	N1—H1B	0.8824
C5—C6	1.480 (2)	N1—H1C	0.8806
C5—C8	1.502 (2)	N2—H2A	0.8805
C6—O6	1.2655 (18)	N2—H2B	0.9002
C6—C7	1.420 (2)	N2—H2C	0.9002
C7—O7	1.2958 (18)	O4—H4	1.0509
C7—C8	1.450 (2)	O7—H7	1.0597
C8—O8	1.2380 (18)	O1W—H1W	0.9203
C9—N1	1.480 (2)	O1W—H2W	0.9287
			
O1—C1—C4	136.62 (14)	C9^i^—C9—H9A	109.7
O1—C1—C2	135.22 (15)	N1—C9—H9B	109.7
C4—C1—C2	88.16 (12)	C9^i^—C9—H9B	109.7
O2—C2—C3	136.56 (15)	H9A—C9—H9B	108.2
O2—C2—C1	134.96 (15)	N2—C10—C10^ii^	110.98 (17)
C3—C2—C1	88.47 (12)	N2—C10—H10A	109.4
O3—C3—C4	133.57 (14)	C10^ii^—C10—H10A	109.4
O3—C3—C2	135.63 (14)	N2—C10—H10B	109.4
C4—C3—C2	90.81 (12)	C10^ii^—C10—H10B	109.4
O4—C4—C3	131.03 (14)	H10A—C10—H10B	108.0
O4—C4—C1	136.44 (14)	C9—N1—H1A	110.3
C3—C4—C1	92.52 (12)	C9—N1—H1B	107.2
O5—C5—C6	136.10 (14)	H1A—N1—H1B	110.0
O5—C5—C8	135.69 (15)	C9—N1—H1C	109.3
C6—C5—C8	88.19 (11)	H1A—N1—H1C	109.7
O6—C6—C7	133.96 (14)	H1B—N1—H1C	110.3
O6—C6—C5	135.43 (14)	C10—N2—H2A	109.2
C7—C6—C5	90.58 (12)	C10—N2—H2B	111.0
O7—C7—C6	131.89 (14)	H2A—N2—H2B	109.8
O7—C7—C8	135.48 (14)	C10—N2—H2C	110.2
C6—C7—C8	92.61 (12)	H2A—N2—H2C	108.5
O8—C8—C7	135.81 (14)	H2B—N2—H2C	108.1
O8—C8—C5	135.57 (14)	C4—O4—H4	111.3
C7—C8—C5	88.59 (12)	C7—O7—H7	110.5
N1—C9—C9^i^	109.73 (16)	H1W—O1W—H2W	106.5
N1—C9—H9A	109.7		

Symmetry codes: (i) −*x*+1, *y*, −*z*+3/2; (ii) −*x*, *y*, −*z*+3/2.

### Hydrogen-bond geometry (Å, °)

**Table:**

*D*—H···*A*	*D*—H	H···*A*	*D*···*A*	*D*—H···*A*
N1—H1A···O5^iii^	0.89	2.14	2.9205 (17)	146.
N1—H1B···O1W^iv^	0.88	1.99	2.8482 (18)	163.
N1—H1C···O8	0.88	1.90	2.7717 (18)	169.
N2—H2A···O2	0.88	1.97	2.8222 (17)	162.
N2—H2B···O1W^v^	0.90	1.94	2.8279 (18)	171.
N2—H2C···O1^iii^	0.90	1.92	2.8071 (17)	168.
O4—H4···O3^vi^	1.05	1.42	2.4675 (15)	179.
O7—H7···O6^v^	1.06	1.41	2.4645 (14)	178.
O1W—H1W···O6	0.92	2.10	2.8724 (17)	140.
O1W—H1W···O8^vi^	0.92	2.40	3.0489 (18)	128.
O1W—H2W···O3	0.93	1.88	2.8035 (19)	171.

Symmetry codes: (iii) *x*, −*y*+1, *z*+1/2; (iv) *x*, *y*+1, *z*; (v) *x*, −*y*, *z*+1/2; (vi) *x*, −*y*, *z*−1/2.
